# Male sex hormones, aging, and inflammation

**DOI:** 10.1007/s10522-022-10002-1

**Published:** 2023-01-04

**Authors:** Justin M. Ketchem, Elizabeth J. Bowman, Carlos M. Isales

**Affiliations:** 1 Medical College of Georgia at Augusta University, Augusta, GA 30912 USA; 2 Augusta University, Augusta, GA 30912 USA; 3 Departments of Medicine, Neuroscience and Regenerative Medicine, Augusta University, Augusta, GA 30912 USA

**Keywords:** Androgens, Hypogonadism, Aging, Inflammation, Sarcopenia, Osteopenia

## Abstract

Adequate levels of androgens (eugonadism), and specifically testosterone, are vital compounds for male quality of life, longevity, and positive health outcomes. Testosterone exerts its effects by binding to the androgen receptor, which is expressed in numerous tissues throughout the body. Significant research has been conducted on the impact of this steroid hormone on skeletal, muscle and adipose tissues and on the cardiovascular, immune, and nervous systems. Testosterone levels have also been studied in relation to the impact of diseases, aging, nutrition and the environment on its circulating levels. Conversely, the impact of testosterone on health has also been evaluated with respect to its cardiac and vascular protective effects, body composition, autoimmunity and all-cause mortality. The male aging process results in decreasing testosterone levels over time. The exact mechanisms and impact of these changes in testosterone levels with age on health- and life-span are still not completely clear. Further research is needed to determine the optimal testosterone and androgen levels to protect from chronic age-related conditions such as frailty and osteoporosis.

## Introduction

In the aging male, there is a loss of overall efficiency and function of many body systems. These functions include changes in overall metabolism, strength, blood pressure, heart rate, appetite, body temperature, sleep/wake cycle, and sexual function. Perhaps the most essential organ system that declines in capacity over time is the endocrine system. The male sex hormones, testosterone and its derivatives, regulate and modulate almost all bodily functions directly or indirectly. Aging has been consistently shown to correlate with marked declines in androgen levels (Harman et al. 2001), which is associated with the development of many chronic and wasting diseases. This review explores current findings regarding androgens, aging, and inflammation. A literature search of PubMed was performed using the following keywords: testosterone, androgens, aging, sarcopenia, osteopenia, and inflammation. Additional searches using the same criteria were performed in the Mendeley and Google Scholar databases. The search was conducted in June and July 2022.

## Production and regulation of testosterone

Testosterone (T) production is regulated by the hypothalamic-pituitary–gonadal (HPG) axis. The hypothalamus releases several tropic hormones with Gonadotropin-Releasing Hormone (GnRH), a peptide hormone, controlling the HPG axis. GnRH is released into the hypothalamic-hypophyseal portal system, which is composed of two connected capillary beds that transport hormones directly from the hypothalamus to the pituitary gland. The pituitary gland is divided into two segments: anterior and posterior. The anterior pituitary is associated with the HPG axis, and GnRH stimulation of gonadotrophin cells results in the release of Luteinizing Hormone (LH) and Follicle Stimulating Hormone (FSH) (Plant and Marshall 2001). These tropic peptide hormones contain two subunits: alpha and beta (Goodman 2004), and act directly on the gonads. In males, FSH acts on the Sertoli cells of the testis to stimulate spermatogenesis (Grinspon et al. 2018). LH acts on the interstitial Leydig cells to stimulate the production and release of testosterone (Choi and Smitz 2014).

GnRH production is significantly increased at the onset of puberty, which results in the release of gonadotropic hormones that stimulate spermatogenesis and testosterone production. Testosterone is responsible for producing and maintaining male secondary sex characteristics, including the deepening of the voice, male hair patterns, anabolic effects, and erythropoiesis. Testosterone is subject to negative feedback to maintain appropriate testosterone levels. Testosterone feeds back and inhibits the release of GnRH by the hypothalamus. Testosterone also functions to inhibit the release of gonadotropins by the anterior pituitary (Winters and Wang 2010).

Testosterone is produced mainly by the smooth endoplasmic reticulum of Leydig cells in the testes. This hormone is derived from cholesterol and is characterized by a hydrophobic structure (Gao et al. 2018). The majority of testosterone is therefore bound to transport and binding proteins in the blood (Clark et al. 2018). These proteins include sex hormone binding globulin (SHBG), human serum albumin (HSA), and to a lesser extent corticosteroid-binding globulin (CBG). A very small percentage of testosterone is unbound in the bloodstream and is known as free testosterone. Total testosterone represents both the bound and unbound concentrations of this hormone (Vankrieken 2000). The free hormone hypothesis postulates that only the unbound fraction of testosterone is active in the target tissues (Laurent et al. 2016a, b). Testosterone exerts its effects by binding to the androgen receptor (AR), a ligand-dependent nuclear transcription factor. The AR is expressed throughout the body in many different tissues including muscle, bone, prostate, adipose, reproductive, cardiovascular, neural, immune, and hematopoietic systems (Davey and Grossmann 2016).

Testosterone is metabolized by two main enzymes: aromatase and 5α-reductase. Aromatase is located in the male gonads, as well as bone, breast, brain, and adipose tissues (Durdiakova et al. 2011). Aromatase catalyzes the conversion of testosterone into estradiol, which binds to estrogen receptors (Stocco 2012). Estradiol has traditionally been considered a female sex hormone, but it also plays vital roles in males by promoting spermatogenesis, libido, and erectile function (Schulster et al. 2016). 5 α-reductase catalyzes the conversion of testosterone into 5 α-dihydrotestosterone (DHT) (Randall 1994). 5 α-reductase is located mainly in the prostate, liver, and skin (Marks 2004). DHT is the most potent androgen, and it binds to the AR with the highest affinity. At the onset of puberty, DHT is primarily responsible for facial and body hair growth in addition to prostate growth (Marchetti and Barth 2013). In adults, DHT has been correlated to male pattern baldness and hair loss (Fu et al. 2021).

Testosterone has been shown to decline with age with up to 50% of men over the age of 80 being classified as hypogonadal (Harman et al. 2001). Androgen levels have been shown to peak at approximately 30 years of age with a steady decline of about 1–2% per year thereafter (Araujo et al. 2004). The mechanism responsible for this age-dependent decrease in androgen levels is not fully known. However, it has been demonstrated that the aging process increases the negative feedback of testosterone on the HPG axis (Winters and Atkinson 1997). Other studies have shown that the number of Leydig cells in the testes of older men are 44% lower than in younger men with a significant negative correlation with age. In addition, serum LH levels are nearly doubled in older men, indicating testicular non-responsiveness and failure to secrete T (Neaves et al. 1984). Another study confirmed these findings by determining that GnRH secretion is lower in older men, but LH secretion is relatively stable (Veldhuis et al. 2012). Higher levels of cytokines have also been shown to downregulate the reproductive axis and decrease GnRH secretion (Igaz et al. 2006). Furthermore, insulin sensitivity has been shown to be critical in the expression and secretion of GnRH (Gamba and Pralong 2006).

**Fig. 1:**
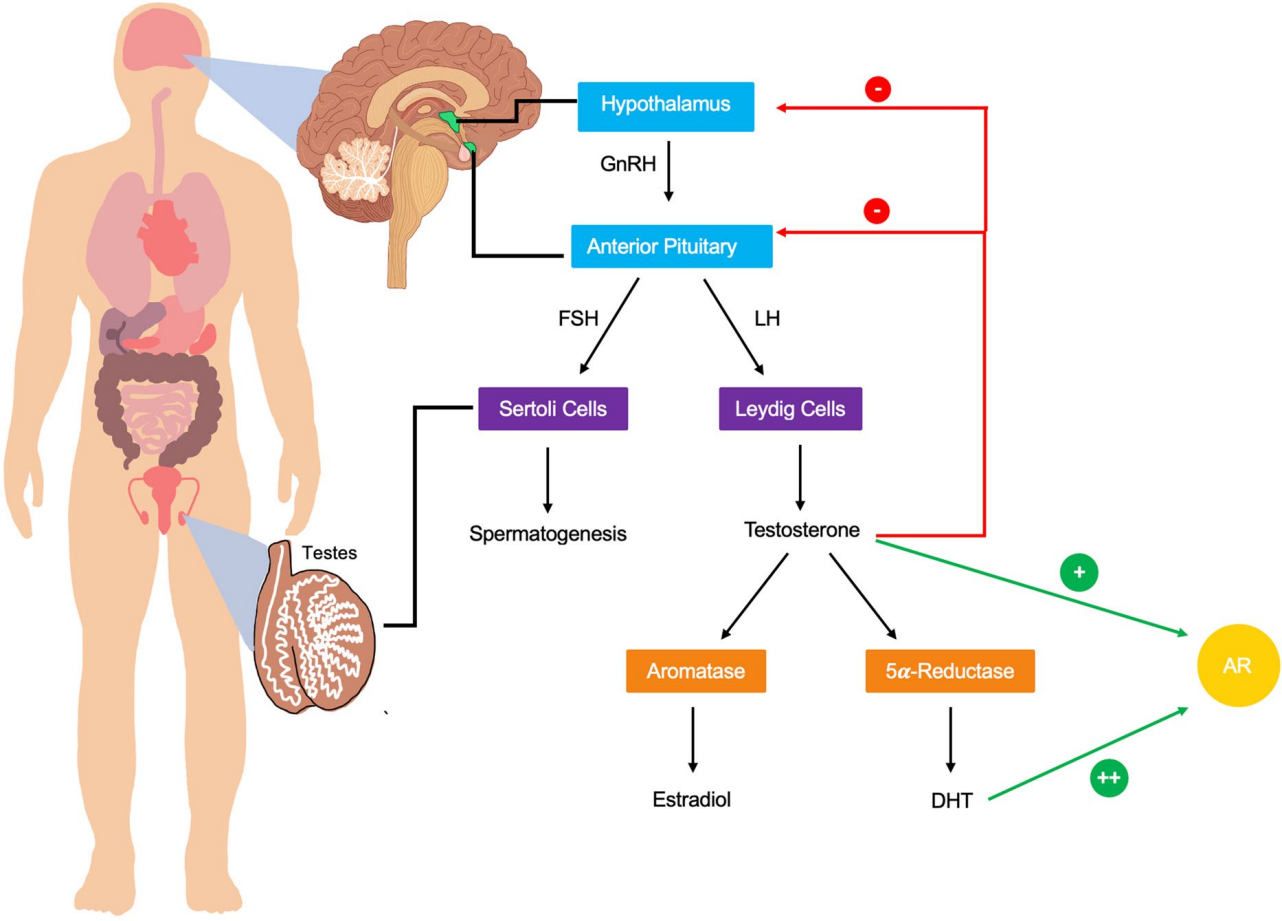
Regulation and metabolism of testosterone. Testosterone production begins in the hypothalamus when GnRH is released into the hypophyseal portal system and acts upon the anterior pituitary causing the release of FSH and LH (Plant and Marshall 2001). FSH acts on the Sertoli cells of the testes to stimulate spermatogenesis (Grinspon et al. 2018). LH acts on the Leydig cells of the testes causing the release of Testosterone (Choi and Smitz 2014). Testosterone is metabolized into DHT by the action of 5α-reductase (Randall 1994). Both testosterone and DHT act on the AR with DHT exerting more potent effects (Marchetti and Barth 2013). Aromatase converts testosterone into estradiol (Stocco 2012) which regulates many bodily processes (Schulster et al. 2016)

A large-scale meta-analysis of 9054 men in the United States and Europe demonstrated the dramatic and age-related decline in total testosterone. This study set the harmonized reference range for healthy nonobese men from 19 to 39 years old at 264 to 916 ng/dL (Travison et al. 2017). Another important conclusion from this study is that some men have a more drastic decline in free T levels as compared to other groups of men. The 95th percentile of men in the 19–39 years old age range has a total T level of 850 ng/dL. The 95th percentile of men in the 80 to 99 years old age range has a total T level of 839 ng/dL (Travison et al. 2017). This finding illustrates that at the high end of the natural total T range, there is very little drop off due to age. In contrast, the 5th percentile of men in the 19 to 39 years old age range has a total T level of 304 ng/dL. The 5th percentile of men in the 80 to 99 years old age range has a total T level of 218 ng/dL (Travison et al. 2017). This massive reduction in total T levels at the low end of the normal range of free T demonstrates that the androgen levels of some groups of men are affected more significantly by aging (Fig. 1).

## Testosterone effects on organ systems

Androgens such as testosterone and DHT exert their actions in a wide variety of tissues by binding to the AR. The AR is located in the cytoplasm, and androgens are able to diffuse through the plasma membrane and bind to the AR due to their hydrophobic nature (Davey and Grossmann 2016). The AR has two distinct pathways of signaling. The first is referred to as the classical or DNA binding dependent pathway, which involves the androgen/AR complex entering the nucleus and binding to androgen response elements (AREs) to modulate gene expression (Eder et al. 2001). The classical pathway is speculated to result in more prolonged effects. The second AR pathway is known as the non-genomic or non-classical pathway, which involves the activation of second messenger cascades including extracellular signal regulated kinase (ERK), Akt, and mitogen activated protein kinase (MAPK) (Estrada et al. 2003). These effects occur much more rapidly than DNA binding dependent signaling and are speculated to be initiated by membrane bound receptors (Papakonstanti et al. 2003).

### Skeleton

Androgens are involved in the maintenance of bone health and the equilibrium between bone breakdown and formation. Osteoblasts are primarily involved in the formation and strengthening of bones, while osteoclasts are involved with bone resorption (Matsuo and Irie 2008). Osteoporotic fractures in men increase significantly at older stages of life and are paralleled by a decrease in bioavailable testosterone (Mohamad et al. 2016). Multiple studies have shown that both testosterone and DHT increase growth and proliferation of osteoblasts (Nakano et al. 1994), as well as suppress osteoblastic apoptosis (Kousteni et al. 2001). Estradiol that has been created by the conversion of testosterone by aromatase has been demonstrated to prevent osteocyte apoptosis (Tomkinson et al. 1997). In addition, DHT has been shown to inhibit the development of osteoclasts and prevent bone resorption (Huber et al. 2001). These effects result in increased bone deposition, maintenance of bone structure, and prevention of bone loss resulting in osteoporosis. Some studies have shown that testosterone administration increases bone mineral density (BMD) in hypogonadal men, and its’ use could also be effective in the treatment of osteopenia (Shigehara et al. 2021).

### Skeletal muscle

Skeletal muscle is also affected by androgens. Testosterone has been shown to increase muscle protein synthesis (MPS) without affecting whole body protein synthesis (Griggs et al. 1989). This increase in MPS may be explained by the increased expression of intramuscular insulin like growth factor 1 (IGF1) (Urban et al. 1995). IGF1 has been shown to increase anabolic pathways and hypertrophy within skeletal muscle (Ascenzi et al. 2019). Synthetic testosterone administration has also been shown to increase the expression of AR on skeletal muscle samples while increasing the utilization of amino acids resulting in anabolism (Sheffield-Moore et al. 1999). In addition, androgens have been shown to prevent the suppression of MPS by glucocorticoids (Hickson et al. 1990). Testosterone administration has been shown to increase fat free mass and maximal voluntary strength in a dose dependent manner (Bhasin et al. 2001). These effects may be explained by research suggesting that androgens interact with the AR to promote the formation of myoblasts as well and inhibit adipogenesis (Herbst and Bhasin 2004).

### Adipose tissue

Androgens further impact body composition via interactions within adipose tissue. Testosterone has been shown to restrain fatty acid (FA) storage by suppressing lipoprotein lipase (LPL) and acyl coenzyme A synthetase (ACS) activity (Santosa et al. 2017). LPL is an extracellular enzyme that functions to break down triglycerides into FAs and glycerol. The free FAs are used for energy storage primarily in adipose tissue (Mead et al. 2002). ACS is required for the transport of FAs into cells, where it begins to activate those FAs by catalyzing the formation of fatty acyl-CoA (Weimar et al. 2002). By decreasing the activity of LPL and ACS, testosterone inhibits lipogenesis and accumulation of adipose tissue. Adipose tissue can be considered a non classical endocrine organ due to its production and release of a number of circulating factors (Kershaw and Flier 2004). One of these factors is leptin, which has been shown in excess to inhibit the production of androgens in the Leydig cells directly. This mechanism of action suggests that obesity may play a crucial role in hypogonadism (Caprio et al. 2001). In addition, stromal cells of adipose tissue contain high concentrations of aromatase, which functions to convert testosterone into estradiol (Cleland et al. 1983). Testosterone administration has also been observed to increase the responsiveness of adipose tissue in addition to decreasing abdominal fat in hypogonadal men (Rebuffe-Scrive et al. 1991).

### Cardiovascular/hematopoietic

The cardiovascular and hematopoietic systems are also functionally affected by androgen signaling. Considerable debate exists over the relationship between endogenous androgen levels and cardiovascular disease (CVD). Some studies have shown that normal endogenous levels of androgen may act in a cardioprotective role in preventing CVD (Turhan et al. 2007). In addition, a meta-analysis of 70 studies demonstrated that CVD patients exhibited significantly lower levels of testosterone and higher levels of estradiol than matched controls (Kloner et al. 2016). Patients with low testosterone were also more likely to exhibit atherosclerotic plaques, endothelial dysfunction, and higher levels of C reactive protein (CRP) indicating an inflammatory response (Farias et al. 2014). The mechanisms involved are still unclear, but the relationship between testosterone and CVD is heavily debated because low testosterone may simply be an indicator of declining total body health (Shores and Matsumoto 2014). Testosterone administration has also been documented to stimulate erythropoietin (EPO) and reduce ferritin and hepcidin levels. Androgens have also been shown to increase the activity of red bone marrow and promote iron incorporation into developing red cells (Shahani et al. 2009). These resultant changes cause hematocrit to increase by approximately 10% (Bachman et al. 2014).

### Immune system

AR has been observed in thymocytes of the immune system (Viselli et al. 1995a, b), and castration or androgen depletion has been shown to lead to enlargement of the thymus (Olsen and Kovacs 2001). The mechanism for this enlargement has been speculated to involve AR mediated thymocyte apoptosis (Olsen et al. 1998) as well as AR induction of downregulatory cytokines, including transforming growth factor beta (TGFβ) (Olsen et al. 1993). AR was also found to be expressed in immature B cells and marrow stromal cells. Androgens were shown to increase the production of functional B cells, with castrated mice accumulating B cells in the bone marrow (Viselli et al. 1997). In a mouse castration model, one study found that androgen deprivation resulted in a decrease in the number of mature T cells and an increase in the production of autoreactive antibodies (Viselli et al. 1995a, b).

Testosterone administration has been demonstrated to decrease the pro-inflammatory actions of macrophages. T administration reduces the expression of toll-like receptor (TLR) 4 (Rettew et al. 2008). Testosterone has also been shown to reduce the expression and secretion of tumor necrosis factor α (TNF-α) and interleukin 1-β (IL-1β), pro-inflammatory cytokines released from macrophages (Corcoran et al. 2010). Dendritic cells (DC) have been shown to decrease the secretion of pro-inflammatory cytokines when treated with testosterone, and the resultant immunosuppressive effects last for up to three months after cessation of therapy (Corrales et al. 2009).

### Nervous system

ARs have been repeatedly detected in the central nervous system. The presence of AR is widespread and has many downstream effects (Roselli et al. 1989). Testosterone has been demonstrated to exert neuroprotective effects against the harmful actions of reactive oxygen species (ROS). This protection can be measured through a decrease in apoptotic cells and an increase in antioxidant catalase activity when cerebral cells are treated with T. This protection was abolished by administration of the AR antagonist flutamide, which suggests that T exerts these protective effects by binding to the AR (Ahlbom et al. 2001). T has also been shown to decrease β-amyloid (Aβ) toxicity in isolated hippocampal neurons. This finding indicates that T may be neuroprotective, and a deficiency of androgen could contribute to the development of Alzheimer’s disease (AD). This study also indicates that T is neuroprotective independent of aromatase or estradiol effects (Pike 2001). Testosterone has also been demonstrated to prevent Aβ pathology by reducing the accumulation of tau tangles, mainly via estrogen pathways (Rosario et al. 2010).

Experimental autoimmune encephalomyelitis (EAE) is used to investigate multiple sclerosis (MS) pathology and potential treatments (Constantinescu et al. 2011). Androgens have been consistently shown to protect against EAE, which may indicate that testosterone supplementation could be beneficial in men with MS (Palaszynski et al. 2004). In mice treated with androgen, this protection may be mediated by enhanced production of the anti-inflammatory cytokine IL-10 (Dalal et al. 1997). These neuroprotective effects have been observed to reverse when subjects are treated with the AR antagonist flutamide, indicating that interaction with the AR is key (Matejuk et al. 2005). It is important to note that estrogen also provides anti-inflammatory and neuroprotective effects in EAE, so the effect of T may be mediated by aromatase activity (Gold and Voskuhl 2009). Through interaction with the AR, androgens have been shown to promote remyelination in mice treated with a demyelinating toxin (Hussain et al. 2013).

Through the actions of 5α-reductase, testosterone is metabolized into DHT, which is neuroactive. DHT has been shown to regulate dendritic spine maturation and long-term potentiation in male rodents. This increase in neural connection can be stopped by administering finasteride, which inhibits 5α-reductase (Brandt et al. 2020). Signaling via AR has been shown to increase the concentration of neural progenitor cells (NPCs) and lead to neurogenesis (La Rosa et al. 2021).

Several conditions of the nervous system are correlated with significant changes in androgens. Low levels of DHT have been observed in male reeler mice (experimental model of autism) at very early stages of growth (Biamonte et al. 2009). Although subjects with traumatic brain injury (TBI) have been observed to have significantly lower DHT levels following injury, this difference is only observed in male mice (Lopez-Rodriguez et al. 2016). EAE has also been observed to be correlated with lower DHT and estradiol levels in the spinal cord of affected mice (Caruso et al. 2010).

**Fig. 2:**
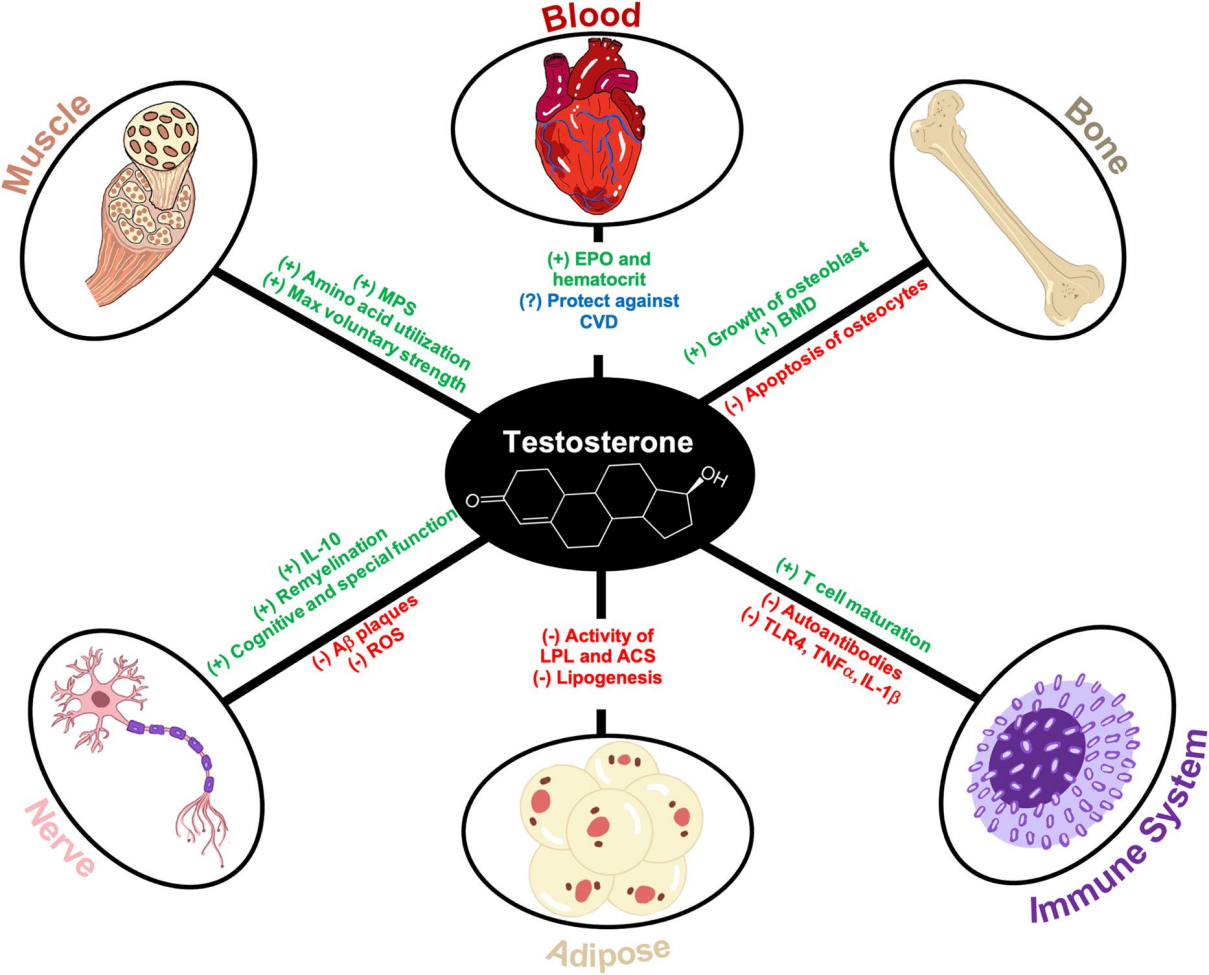
Androgen effects on organ systems. Testosterone and its derivatives have been shown to result in increased muscle protein synthesis (Griggs et al. 1989), amino acid utilization (Sheffield-Moore et al. 1999), and maximum skeletal muscle strength (Bhasin et al. 2001). Androgens have been shown to increase EPO and hematocrit with their effects on CVD still under debate (Shores and Matsumoto 2014). Testosterone increases BMD and osteoblast growth (Nakano et al. 1994) while concurrently decreasing apoptosis of osteocytes (Kousteni et al. 2001). Testosterone increases T cell maturation (Viselli et al. 1995a, b), decreases the proliferation of autoantibodies (Rettew et al. 2008), as well as signaling involving TLR4, TNF-α, and IL-1β (Corcoran et al. 2010). Androgens decrease the activity of LPL and ACS (Santosa et al. 2017), resulting in a decline in lipogenesis (Weimar et al. 2002). The nervous system responds to testosterone by increasing ant-inflammatory markers including IL-10 (Dalal et al. 1997), remyelination of neurons (Palaszynski et al. 2004), and increasing cognitive and spatial function (Azad et al. 2003; Barrett-Connor et al. 1999; Janowsky et al. 1994). Androgens also decrease the concentration of ROS (Ahlbom et al. 2001) and Aβ plaques in the nervous system (Pike 2001)

Testosterone administration has been shown to enhance blood flow to the midbrain and superior frontal gyrus. This effect may explain the improvement in cognitive function that some patients experience with testosterone replacement therapy (TRT) (Azad et al. 2003). In older men, low estradiol and high testosterone levels predict better performance on several tests of cognitive function (Barrett-Connor et al. 1999). TRT has also been linked to an increase in the spatial cognition of older men (Janowsky et al. 1994) (Fig. 2).

## Testosterone effects on illness/disease

Androgens have been consistently demonstrated to affect a wide range of illnesses and disease pathologies. In this review, we focus specifically on hypogonadism as a contributing or exacerbating factor of a number of disease states.

### Mental illness/well-being

Low T levels have been shown to be associated with increased incidence and earlier onset of depressive illness. This correlation has been shown to be even more vital for men aged 50 to 65 and men with high medical morbidity (Shores et al. 2005). A meta-analysis determined that testosterone may have an antidepressant effect, especially in elderly patients and patients with human immunodeficiency virus (HIV) or acquired immunodeficiency syndrome (AIDS) (Zarrouf et al. 2009). Testosterone and its derivative (DHT and estradiol) have been documented to enhance the activity of dopaminergic neurons in the mesolimbic system, which may explain the increased sense of well-being on TRT (Alderson and Baum 1981). Another study revealed that the administration of estrogen or testosterone resulted in an increase in the firing activity of serotonergic neurons in the dorsal raphe nucleus of rats (Robichaud and Debonnel 2005). Recent studies have found that the mental impacts of testosterone may be primarily mediated by the MAPK pathway of the hippocampus (McHenry et al. 2014). TRT has been demonstrated to significantly decrease ratings of anger, irritability, sadness, tiredness, and nervousness. TRT also significantly improved ratings of energy level, friendliness, and sense of well-being. Similar relationships were observed for DHT and mood ratings (Wang et al. 1996). DHT has been noted to become further reduced into neurosteroids that are potent γ-aminobutyric acid class A (GABA_A_) agonists. The activation of GABA_A_ results in anxiolytic effects and may be implicated in anxiety disorders (Aikey et al. 2002).

### Body composition

Testosterone has been shown to increase fat free mass, muscle size, and strength when given to older men and HIV infected men with weight loss (Bhasin et al. 2001). In men treated with glucocorticoids for a long duration of time, testosterone levels have been shown to decline significantly. In addition, these patients were noted to have increased levels of muscle wasting and loss of bone density (Reid et al. 1985). Testosterone therapy has been shown to increase muscle strength, body composition, quality of life and physical function in older men who are classified as frail (Srinivas-Shankar et al. 2010). Further studies have shown that intramuscular (IM) testosterone administration increases lumbar BMD by 8%, which could the development of osteoporosis and future fractures (Tracz et al. 2006).

Testosterone has been shown to increase lean body mass and mid arm circumference and to also decrease the waist to hip ratio in elderly men with decreased testosterone levels (Vermeulen et al. 1999). Randomized control trials (RCTs) have found that testosterone therapy has a positive effect on body composition and increases BMD in hypogonadal men (Yeap 2009). Testosterone treatment increases the quality of life and mental health in HIV + patients (Knapp et al. 2008). Chronic obstructive pulmonary disease (COPD) has also been correlated with low T levels and muscle weakness. Testosterone administration has been shown to increase lean body mass (LBM) and one repetition strength. As tested, these benefits were even more pronounced when combined with a resistance training routine (Casaburi et al. 2004).

The combination of testosterone and nutritional supplementation reduced the hospitalization rates and the duration of hospital stays in elderly patients with defined malnutrition (Chapman et al. 2009). Oxandrolone, a DHT derivative, has been used to treat many wasting diseases and to increase wound healing (Zhao et al. 2004). In addition, oxandrolone is the only anabolic androgenic steroid (AAS) that is FDA approved for the treatment of weight loss after severe trauma, major surgery, infection, malnutrition, and muscular dystrophy (Orr and Singh 2004). Oxandrolone has been documented to increase protein synthesis efficiency, decrease protein catabolism, and increase wound healing in severe burn patients (Hart et al. 2001; Demling and Orgill 2000). Oxandrolone has also been shown to increase body cell mass (BCM) and quality of life in HIV infected patients (Earthman et al. 2002). Further studies support the use of oxandrolone in HIV + patients, with significant improvements in the weight and well-being of patients suffering from wasting and weakness. In addition, oxandrolone was well tolerated by all patients in these studies (Berger et al. 1996).

### Fighting infection

Hypogonadism is one of the most common associated effects of HIV infection. In most cases, this deficiency is caused by low levels of gonadotrophins (Poretsky et al. 1995). Endocrine disorders have been well documented in patients with AIDS, with some studies reporting that 50% of AIDS patients are classified as hypogonadal (Dobs et al. 1988).

Dehydroepiandrosterone (DHEA) and DHEA-sulfate (DHEA-S) are androgens that are produced by the zona reticularis of the adrenal cortex. These compounds do not activate the AR but can be peripherally converted into testosterone and DHT (Auchus and Rainey 2004). Lower levels of DHEA-S are correlated with advanced progression of AIDS, low CD4 count, and high HIV RNA. In addition, free T was inversely correlated with HIV RNA levels (Ferrando et al. 1999). Feline studies have demonstrated that DHEA administration decreased inflammatory gene transcripts while promoting CD4 + T cell production and neuronal preservation (Maingat et al. 2013).

In the recent COVID-19 pandemic, testosterone has been suspected of playing a protective role in the progression of severe acute respiratory syndrome (SARS-CoV-2) infection to the development of acute respiratory distress syndrome (ARDS). In a study of hospitalized male COVID-19 patients, testosterone levels were inversely correlated with IL6, C reactive protein (CRP), and interferon γ (IFN-γ) inducible protein 10. Also, men with lower T levels were much more likely to have a severe disease progression (Dhindsa et al. 2021). In addition, SARS-CoV-2 has been demonstrated to bind via the angiotensin-converting enzyme II (ACE2). This membrane-bound protein has also been shown to be constitutively expressed in adult Leydig cells (Douglas et al. 2004). This relationship suggests that SARS-CoV-2 may alter the ability of the testis to produce or secrete testosterone (Hackett and Kirby 2020). Testosterone levels have been consistently observed as significantly lower in critically ill male COVID-19 patients. No perceptible difference in sex hormones was observed in female patients. In addition, male patients with higher estradiol levels were more likely to receive extracorporeal membrane oxygenation (ECMO) (Schroeder et al. 2021).

Recently, a large amount of data has been evaluated to determine if androgen levels have any relationship to infection and progression of COVID-19. It has been speculated that low testosterone levels are beneficial in preventing the initial infection of SARS-CoV-2 due to blocking transmembrane serine protease 2 (TMPRSS2) to prevent viral entry and blunt the immunosuppressive effects of androgens (Salciccia et al. 2021). A study by Montopoli et al. found that androgen deprivation therapy (ADT) patients had lower rates of infection with SARS-CoV-2 than matched controls (Montopoli et al. 2020). A conflicting analysis of this data determined that ADT was correlated with an increase in lethality from 13 to 25% in men above 70 (Caffo et al. 2020). In terms of disease severity, low T levels can predict ICU transfer and mortality risk in men afflicted with COVID-19 (Rastrelli et al. 2021). Another study demonstrated that total T levels were significantly lower in patients with ARDS than in matched patients without ARDS. Higher serum T levels were also correlated with decreased risk of hypo oxygenation (Salciccia et al. 2020). It is speculated that testosterone serves a protective role in the later stages of infection by suppressing the cytokine storm that is observed in disease progression (Salciccia et al. 2021).

Male mice models have demonstrated that normal gonadal function and physiological T levels protect against influenza infection and progression. Removal of gonads in young males resulted in increased morbidity, illness, and pulmonary pathology. Treatment of gonadectomized males with testosterone resulted in an improvement in all these metrics. T administration increased survival rates, and low T was shown to correlate with adverse outcomes (vom Steeg et al. 2016).

### Cardiac and vascular protection

Testosterone has traditionally been viewed as deleterious to the cardiovascular system. This conclusion is generally based on older trials and data from the abuse of AAS in male athletes. More recent analyses have shown that TRT to a normal physiological range has not been reported to increase the risk of CVD (Iii et al. 2012). A meta-analysis revealed that men with low T levels have reduced survival and increased risk of cardiovascular mortality compared to men with normal or high T levels (Jones 2010). When the AR was knocked out (ARKO) in a strain of mice, atherosclerosis increased significantly. Testosterone administration reduced plaque formation in both the ARKO and wild type (WT) mice. This finding suggests that T has athero-protective qualities via AR dependent and AR independent pathways (Bourghardt et al. 2010). Testosterone levels have been extensively shown to be inversely related to blood pressure (Barrett-Connor and Khaw 1988; Khaw and Barrett-Connor 1988; Hughes et al. 1989; Phillips et al. 1993). Low testosterone has also been shown to be correlated with low levels of antioxidants and increased ROS which may lead to vascular damage (Mancini et al. 2008). In addition, low T levels are associated with erectile dysfunction (ED), which has been recently shown to indicate atherosclerosis and possibly predict a cardiovascular event (Jackson 2012). Testosterone has also been shown to increase the production and release of endothelium-derived nitric oxide (NO). This compound results in vasodilation and may decrease the risk of hypertension (Miller and Mulvagh 2007).

A large cross-sectional study of Chinese men discovered that low total T is a risk factor for the development of hypertension. This study claims that primary hypogonadism should be closely monitored, and this condition places patients at a significantly higher risk of developing CVD (Qu et al. 2021). Numerous studies also support the conclusion that testosterone deficiency is an indicator of CVD and early death. A debate exists in response to the claim that TRT has numerous cardiovascular benefits. Further long term study is required (Jones 2010).

Atherosclerosis has been extensively linked to vascular inflammation. TRT has been significantly shown to reduce pro-inflammatory markers such as TNFα and IL-1β. An increase in the anti-inflammatory marker IL-10 was noted to correlate with a significant reduction in total cholesterol (Malkin et al. 2004). T administration has also been shown to inhibit activation of nuclear factor kappa B (NF-kB), which is required for the expression of vascular cell adhesion molecule 1 (VCAM-1). VCAM-1 is critical in leukocyte adhesion and the early events of atherosclerosis. This pathway suggests that T inhibits the initiation of atherosclerosis (Hatakeyama et al. 2002). Testosterone has been shown as cardioprotective in many studies; however, it is important to understand that T acts through many genomic and non-genomic pathways in addition to its conversion to estradiol. These numerous mechanisms make it difficult to pinpoint the therapeutic benefits of sex hormones (Kelly and Hugh Jones 2013).

### Metabolic syndrome

Metabolic syndrome is classified by excess waist circumference, blood pressure, glucose level, triglycerides, and deficient levels of high density lipoprotein (HDL) cholesterol (Punthakee et al. 2018). Cross sectional studies have demonstrated that low T levels and increased high sensitivity C reactive protein (hs-CRP) are independent predictors of metabolic syndrome (Wickramatilake et al. 2015). This data supports the conclusion that systemic inflammation and endocrine disruption may lead to worsened health outcomes.

A sedentary lifestyle and excessive food consumption are the leading causes of obesity and type 2 diabetes (T2D). These factors are also significant risk factors for hypogonadism. Therefore, there is a complex interplay and some difficulty in determining the cause and effect relationship between hypogonadism and metabolic syndrome (Fink et al. 2018). The adipose tissue aromatase hypothesis holds that an increase in adipose tissue is coupled with an increase in the amount/activity of aromatase. This enzyme converts T into estradiol, which causes negative feedback on the anterior pituitary. This feedback results in lower secretion of gonadotrophins, such as LH, which leads to lower T levels (Fui et al. 2014).

Testosterone has been shown to decrease the number of apoptotic β cells in rat pancreas models. Castrated animals exhibited significantly higher rates of β cell apoptosis that could lead to insulin dependent diabetes mellitus. In addition, administration of an AR antagonist, flutamide, reversed the protection offered by T and resulted in β cell apoptosis. These findings suggest that T preserves insulin secretion by protecting β cells in an AR dependent manner (Morimoto et al. 2005). Further studies have demonstrated that the AR competes with the glucocorticoid receptor (GR) to inhibit apoptosis of pancreatic β cells. These results confirm another mechanism by which androgens protect insulin secretion and sensitivity (Harada et al. 2015).

Androgens have been well documented to reduce the accumulation of adipose tissue through multiple mechanisms. T inhibits lipid uptake and the activity of low-density lipoprotein (LDL) and stimulates lipolysis by increasing the number of β adrenergic receptors. T also reduces the production of leptin, which causes hunger, and inhibits adipocyte differentiation. DHEA has also been observed to increase resting metabolic rate (RMR) and increase the breakdown of lipids by oxidation (De Pergola 2000). T has also been shown to have a more pronounced effect on visceral fat rather than subcutaneous fat, with the highest effects on intra-abdominal adipose stores (Mårin et al. 1996).

The San Antonio Heart Study provides evidence that total T, free T, and DHEA-S were significantly inversely associated with insulin concentrations (Haffner et al. 1994). These relationships provide further evidence that androgens work to promote insulin sensitivity and may prevent the development of T2D. Additional cross-sectional studies have shown that low T levels are significantly correlated with metabolic syndrome in adult males (Hejrati et al. 2020). TRT in physiological ranges has been shown to improve insulin resistance, obesity, dyslipidemia, and quality of life (Muraleedharan and Jones 2010).

### Autoimmune diseases

A large cohort study by Baillargeon et al. determined that a diagnosis of hypogonadism is associated with a significantly higher risk of developing rheumatic autoimmune disease, such as rheumatoid arthritis or lupus (Baillargeon et al. 2016). Testosterone administration has been shown to increase the differentiation of regulatory T cells, which act to suppress the immune response and promote self-tolerance (Fijak et al. 2011). Other studies have shown that an increased E_2_/T ratio is associated with an increased risk of autoimmune thyroid disease (AITD) in males (Chen et al. 2017a, b). The relationship between the microbiota, androgen production, and autoimmune conditions has also been studied. A mouse model demonstrated that male gut microbiota elevates T, reduces islet inflammation, and reduces autoantibody production. These findings point toward T having a protective role in type 1 diabetes (T1D) (Markle Janet et al. 2013). Total T has also been implicated in the development and severity of psoriasis. A case control study demonstrated that severe psoriasis is associated with low levels of serum T (Jean-Pierre et al. 2019). Crohn’s disease has also been shown to improve following the normalization of androgen levels to physiological ranges (Bianchi 2019). The effects of androgens on the development and progression of autoimmune MS have been described in detail above. In addition, T administration has been shown to increase synaptic transmission and protein levels within the hippocampus (Ziehn et al. 2012).

### All-cause mortality

In a longitudinal study of adult US men, low free and bioavailable T were associated with an increased risk of all-cause and cardiovascular mortality. Low SHBG was also associated with a decreased risk of cardiovascular death (Menke et al. 2010). Further meta-analyses have demonstrated similar results with low T associated with increased all-cause mortality and CVD death (Araujo et al. 2011). A retrospective analysis of male veterans determined that men with low T levels had a significantly higher mortality rate than men with normal T levels (Shores et al. 2006). In a comparison study between hypogonadal men either treated with TRT or untreated, the TRT treatment group had a significantly decreased risk of death compared to the untreated group (Shores et al. 2012).

## Inflammation and androgens

Testosterone has been shown to have many anti-inflammatory and protective effects through multiple pathways and mechanisms. T was shown to decrease pro-inflammatory markers such as TNF-α, IL-6, IFN-γ, and IL-2 (Fijak et al. 2011). One of these pathways involves T inhibiting NF-kB signaling resulting in inhibition of p65 signaling (Wang et al. 2021). NF-kB and p65 have been implicated in the production of pro-inflammatory cytokines and the pathogenesis of many inflammatory diseases (Giridharan and Srinivasan 2018). The NF-kB pathway is triggered by TLRs, TNF, and IL-1 (Chen et al. 2017a, b). Androgens have also been shown to inhibit the production and secretion of IL-6 (Gordon et al. 2001). IL-6 has been shown to cause pro-inflammatory effects via the JAK-STAT (Janus kinase/signal transducer) pathway (Chen et al. 2017a, b). Inflammatory cytokines have been observed to result in apoptosis via JAK-STAT signal transduction (Rawlings et al. 2004).

Testosterone has also been observed to decrease the proliferation and activity of group 2 innate lymphoid cells (ILC2), which stimulate the production of inflammatory cytokines (Cephus et al. 2017). A recent meta-analysis determined that testosterone may possess anti-inflammatory effects in vivo due to consistent data supporting that T deficiency is associated with increased inflammatory cytokines (IL-6, IL-1β, and TNF-α) and that T supplementation reduces inflammatory cytokines. In addition, T has been shown to increase anti-inflammatory cytokines such as IL-10 (Mohamad et al. 2019).

Maggio et al. has argued that pro-inflammatory states have a causal relationship to low T production and secretion. IL-6, TNF-α, and IL-1β influence both the HPG axis and testicular function (Maggio et al. 2005). Testosterone has also been observed to act directly on CD4 + T lymphocytes via the AR to increase the production of IL-10 (anti-inflammatory). DHT application was also shown to decrease IL-4, IL-6, IL-12, and IFN-γ levels (Liva and Voskuhl 2001). T has also been shown to decrease the expression and sensitivity of toll-like receptor 4 (TLR4) on isolated macrophages in a mouse model. Orchiectomized mice had significantly higher levels of TLR4 surface expression and were more susceptible to endotoxin shock (Rettew et al. 2008). TLRs have been shown to result in the activation of NF-kB and activator protein-1 (AP-1), which result in inflammation (Chen et al. 2017a, b).

**Fig. 3:**
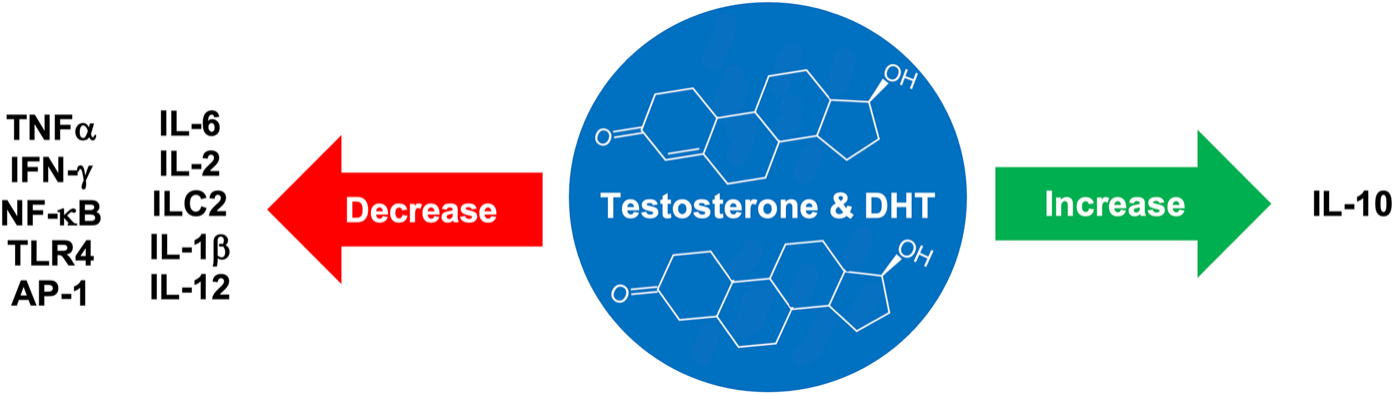
Androgen effects on inflammation. The androgens have been shown to decrease concentrations of inflammatory markers including TNFα, IFN-γ, IL-6, IL-2 (Fijak et al. 2011), NF-κB, AP-1 (Chen et al. 2017a, b), TLR4 (Rettew et al. 2008), ILC2 (Cephus et al. 2017), IL-1β (Mohamad et al. 2019), IL-12. Androgens also increase the concentration of the anti-inflammatory cytokine IL-10 (Liva and Voskuhl 2001)

Androgens have been shown to activate phosphatidylinositol-3 kinase (PI3K), which phosphorylates and activates Akt and downstream mTOR (Basualto-Alarcón et al. 2013). mTOR has been shown to regulate the production of 40S ribosomal subunits, translation initiation and elongation of proteins, and generation of initiation complexes that result in muscular hypertrophy and muscle protein synthesis (Ma and Blenis 2009). These claims have been validated by quantitative studies that reveal that the level of activated mTOR is dependent on the concentration of testosterone (White et al. 2013) (Fig. 3).

### Aging and inflammation

Chronic inflammation is characterized by macrophage infiltration and high levels of circulating pro-inflammatory chemicals (Sarkar and Fisher 2006). The increase in inflammation seen in older men “inflamm-aging” has many different and complex causes. Aging has been shown to be associated with significant increases in ROS (Vigneron and Vousden 2010). ROS damage cells by fragmenting proteins, causing single stranded breaks in DNA and inducing peroxidation of lipids (Farber 1994). These ROS have been shown to result in increased production of NF-kB and downstream inflammation (Ungvari et al. 2007). NF-kB primarily exists as a heterodimer with a p65 and a p50 subunit. NF-kB mainly acts to activate transcription of TNF-α, IL-1, IL-6, IL-8, IFN-γ, and other pro-inflammatory markers (Barnes and Karin 1997). The level of inflammatory markers has been consistently shown to represent a strong risk factor for frailty, disability, and CVD in older men (Ferrucci et al. 2005; Maggio et al. 2006; Stenholm et al. 2010). Inflammation has also been shown to influence a loss of muscle mass and strength in aging men (Schaap et al. 2006).

Another cause of inflammation is the buildup of cellular debris or aggregation of antibodies. The accumulation of these materials can lead to activation of the innate immune system and mitochondrial damage (Sanada et al. 2018). Another explanation for the chronic inflammation in older men is a marked reduction in protective microbiota. Elderly men have been observed to have significantly lower gut microbiome diversity than young adults (Kinross and Nicholson 2012; Claesson et al. 2011). Some of these bacterial species such as Bifidobacterium have been documented to inversely correlate with inflammatory cytokines such as TNF-α and IL-1β. Furthermore, older men are more likely to have increased levels of inflammatory microbiota (Toward et al. 2012).

**Fig. 4:**
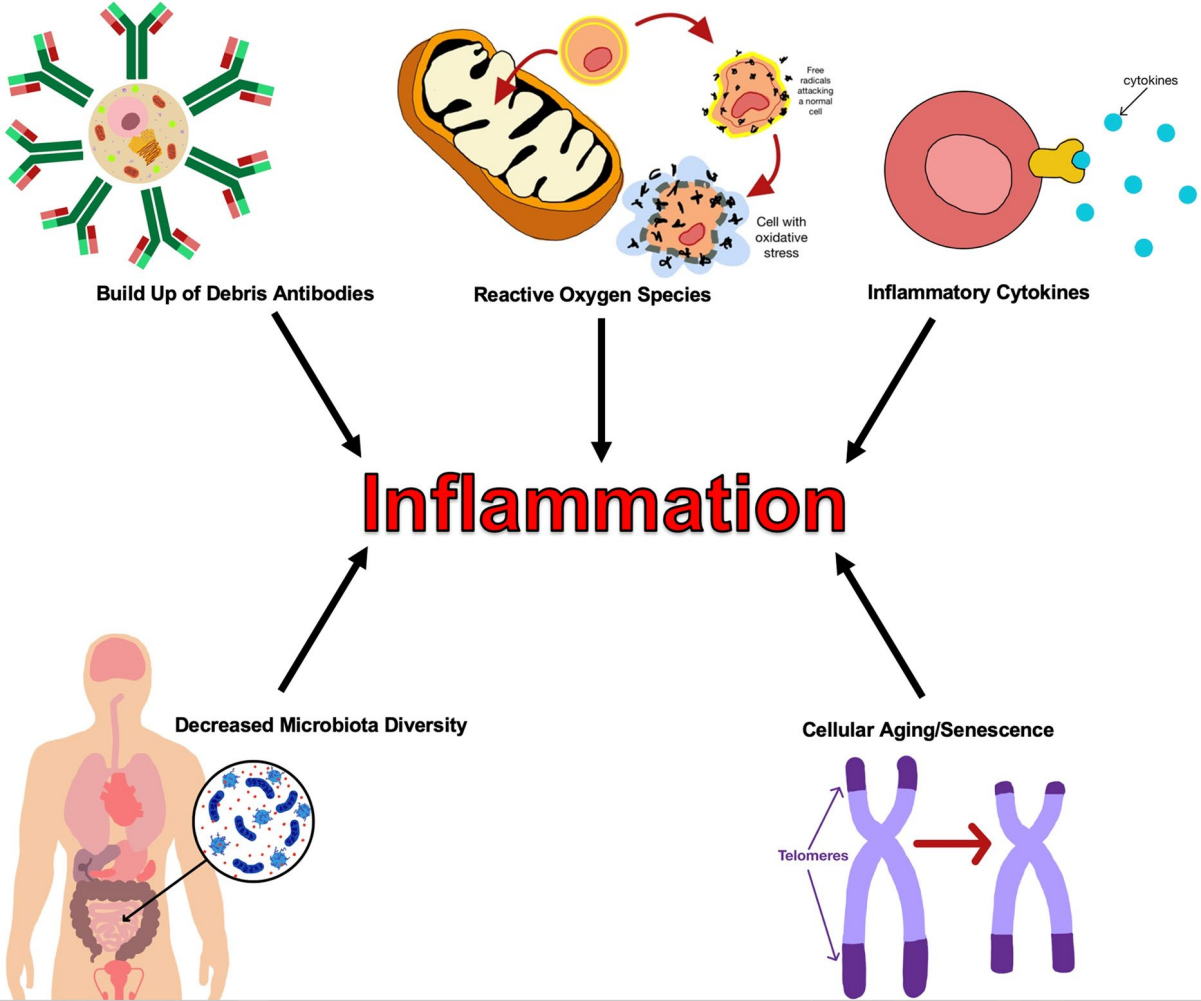
Causes of chronic inflammation. Chronic inflammation has multiple components including a buildup of cellular debris and antibodies (Sanada et al. 2018), elevated levels of ROS (Vigneron and Vousden 2010), increased circulating inflammatory cytokines (Sarkar and Fisher 2006), cellular aging and telomere shortening (Sanada et al. 2009), as well as decreased microbiome diversity (Kinross and Nicholson 2012; Claesson et al. 2011)

Cellular aging and senescence also lead to the release of inflammatory cytokines. The number of senescent cells increases with age, and these cells are classified as the senescence-associated secretory phenotype (SASP). SASP cells have been theorized as the leading cause of several age-related diseases (Sanada et al. 2009; Tchkonia et al. 2013; He and Sharpless 2017). Further animal models have shown that the clearance and reprogramming of SASP cells halt the progression of multiple age-related disorders (Childs Bennett et al. 2016; Jeon et al. 2017; Baker et al. 2011). Immunosenescence or the loss of immune system regulation is characterized by extensive and chronic inflammation (Shaw et al. 2013). This dysregulation increases with age and can result in autoimmunity and increased susceptibility to infections (Aw et al. 2007; Gruver et al. 2007) (Fig. 4).

### Sarcopenia

Sarcopenia, or an age-related loss of muscle mass and force, generally begins at approximately 50 years of age. This loss begins with a 1% loss per year and then accelerates at 70 years of age. Approximately 50% of individuals over the age of 80 can be diagnosed with sarcopenia (Morley et al. 2014). Sarcopenia has been shown to impair daily movement and activity (Janssen et al. 2002), lead to falls and injuries, and significantly increase the risk of respiratory failure in the elderly (Landi et al. 2013). Patients with sarcopenia are significantly more likely to be immobile and hospitalized than patients with healthy muscle tissue (Welch et al. 2018). Sarcopenia can also be linked to excess inflammation leading to apoptosis of muscle cells. TNF-α has been shown to be responsible for skeletal muscle loss by causing myocyte apoptosis in myocytes. TNF-α has been observed to activate caspase-3 via caspase 8 and result in apoptosis (Phillips and Leeuwenburgh 2005). NF-kB induces multiple pro-inflammatory pathways that can result in the loss of skeletal muscle. Inhibitors of NF-kB have been demonstrated to attenuate body weight loss and prevent catabolism of skeletal muscle (Cai et al. 2004). Sarcopenia has also been shown to increase with declining skeletal muscle mitochondria number and quality (Romanello 2021).

Inflammation has been shown to cause damage to skeletal muscle (Anker et al. 1999). The mechanisms by which inflammation can lead to sarcopenia are still being researched. Inflammation has been shown to drastically reduce IGF-1, which acts as a mediator for growth hormone (GH). A deficiency of IGF-1 has been linked with sarcopenia and frailty (Roth et al. 2006). Pro-inflammatory cytokines have also been observed to correlate with anorexia of aging and may cause nutrient deficiencies that lead to sarcopenia (Bales and Ritchie 2002). Muscle protein synthesis requires many nutrients that are signaled by the mammalian target of rapamycin (mTOR) and AMP activated protein kinase (AMPK) (Fujita and Nonomura 2019). The increase in autoimmunity with aging could also contribute to the catabolism of skeletal muscle (Chung et al. 2009). Levels of free T have been shown to inversely correlate with limitations in mobility. Men with low free T were 68% more likely to experience mobility limitation than men with normal free T levels. This correlation did not exist for total T or SHBG (Krasnoff et al. 2010). The mechanisms by which androgens increase muscular size and strength are still unclear, however androgens have been shown to stimulate protein synthesis by short term and long term mechanisms that increase ribosome quantity (Isidori et al. 2005).

### Osteopenia

Osteopenia results from an age-related loss in bone mass that can lead to decreased mobility and quality of life (McCormick 2007). Chronic inflammation has been shown to disrupt bone homeostasis and may increase the catabolism of bone via osteoclasts (Chung et al. 2009). Increased systemic levels of CRP have been observed to correlate with lower BMD (Rittayamai et al. 2012). Androgen administration has been shown to suppress trabecular bone loss and body weight loss in a botulinum toxin (BTx) mouse model. In addition, mice with AR knockout in osteocytes and satellite cells had significantly higher bone loss than control subjects (Laurent et al. 2016a, b). These studies suggest that androgens, via an AR dependent mechanism, act to prevent bone resorption and may play a role in mitigating osteopenia. In a longitudinal study following patients with androgen insensitivity, all five of the included adults developed significant osteopenia in their lumbar spine and femoral neck (Soule et al. 1995). This study shows that AR-mediated effects are vital for healthy skeletal development and may indicate that androgen therapy may benefit patients at risk of osteopenia. A prospective study of 200 older male participants determined that 70% of men with osteoporosis also exhibited low serum T levels (Kotwal et al. 2018).

## Aging and androgens

Many different factors can contribute to the decrease in testosterone levels throughout a man’s life. Androgens have been consistently shown to decrease with age. The Baltimore Longitudinal Study on Aging found that the incidence of total T hypogonadism was 20% over age 60 increasing to 30% over age 70 and then to 50% of men over age 80. An even higher drop-off in free T levels was also observed in this study (Harman et al. 2001). Further cross-sectional studies have also shown that total T and estradiol levels decrease significantly with age (Ferrini and Barrett-Connor 1998). Furthermore, the level of free or bioavailable T has been shown to decline more dramatically than total T. This change has been linked to an increase in SHBG in older men. The Massachusetts Male Aging Study found that SHBG increased by 1.6% per year after the age of 40. Total T dropped by 1.6% per year and free T dropped by 2–3% per year (Feldman et al. 2002). In addition, several studies have shown that muscle mass and voluntary strength decrease with age. Elderly subjects were found to have 26% lower cross-sectional area and maximum voluntary force of their skeletal muscle (Phillips et al. 1992).

The dual defect hypothesis states that a bodily failure of both the hypothalamic-pituitary axis and Leydig cells are responsible for the age-related decrease in androgen levels. The number of Leydig cells and their functional ability to secrete T declines with age (Haji et al. 1994). A rodent model demonstrated that Leydig cells of older subjects had lower levels of steroidogenic acute regulatory (StAR) protein, representing a decline in the ability to produce and release androgens (Leers-Sucheta et al. 1999). StAR protein is required to transport cholesterol and steroid hormones through the inner mitochondrial membrane where these compounds can be processed into androgens (Manna et al. 2016). The release of GnRH has been shown to decline markedly in older men, resulting in lower pulses of LH secretion (Keenan et al. 2006). GnRH secretion has been observed to decrease consistently with age when compared to young men (Pincus et al. 1997). Older men have been shown to have a significantly lower response to GnRH as compared to younger men, representing a deficiency in Leydig cell function (Korenman et al. 1990), (Harman and Tsitouras 1980). Further studies have shown lower secretion of LH in older men (Veldhuis et al. 1999). Rodent studies have also described a decline in the number of AR in older subjects (Haji et al. 1981). Older men also have a weakened response to LH when compared to younger men. After administration of recombinant LH, older men had a 50% lower T secretion response (Veldhuis 2008).

Primary hypogonadism is diagnosed by low serum T levels coupled with elevated LH levels indicating dysfunction of the testis (McBride et al. 2015). One main cause of primary hypogonadism is varicocele, which increases in occurrence significantly with advanced age. A recent study demonstrated that 42% of the elderly male population exhibited varicocele (Canales et al. 2005). A varicocele is an enlargement of the veins that return blood from the scrotum. Varicocele has been shown to be significantly correlated with lower T levels in men than age matched controls. Further, when these men underwent surgery to repair the varicocele, 70% of patients experienced a significant increase in testosterone with a mean improvement of 178 ng/dL (Tanrikut et al. 2011).

The secretory capacity of Leydig cells has also been shown to decrease with age, specifically with the number of Leydig cells (Neaves et al. 1985). The ability of the testes to produce androgens also declines with age. The steroidogenic enzymes have been shown to decrease in number and activity with age. In addition, the transport of cholesterol into testicular mitochondria is also increasingly impaired in older men (Zirkin and Chen 2000; Culty et al. 2002). Testicular volume has also been shown to decrease dramatically with age. One study found that testicular volume was reduced by 30% compared to young men in a cohort of men over age 75 (Mahmoud et al. 2003).

**Fig. 5:**
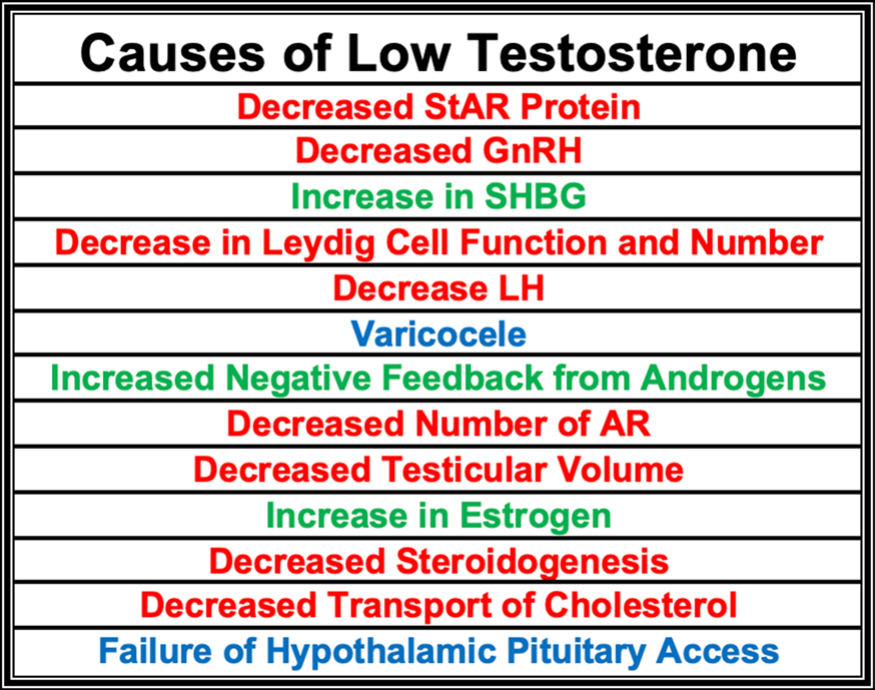
Causes of Decline in Androgen Levels with Age Androgen levels decline with age due to many factors including decreased StAR protein (Manna et al. 2016) and secretion of GnRH (Keenan et al. 2006). Levels of SHBG have been observed to increase at a dramatic rate in older men (Feldman et al. 2002). Other contributing factors include a decline in Leydig cell number and function (Korenman et al. 1990), as well as LH levels (Veldhuis et al. 1999). Androgen levels also decline with the occurrence of varicocele (Tanrikut et al. 2011), negative feedback on the HPG axis (Pincus et al. 1997), a decline in the concentration of AR (Haji et al. 1981), decreased testicular volume (Mahmoud et al. 2003), and increased estrogen levels (Ferrini and Barrett-Connor 1998)

Secondary hypogonadism is typically due to decreased gonadotrophin production (McBride et al. 2015). Older men have been observed to have increased sensitivity to negative feedback from androgens (Deslypere et al. 1987; Winters et al. 1984; Mulligan et al. 1997; Winters and Atkinson 1997). This sensitivity is still under investigation and is not fully understood from a medical standpoint (Fig. 5).

## Discussion

Testosterone and its derivatives have been shown to interact with the AR to activate genomic and non-genomic pathways that affect a wide variety of body tissues. The evidence presented in this review indicates a variety of conditions that may be correlated with and possibly caused by low levels of androgens. Healthy levels of androgens, or eugonadism, have been demonstrated to correlate with a higher quality of life, better longevity, and positive health outcomes. Androgen levels have also been shown to consistently decline in the aging male. Hypogonadism is of greater importance in older men, and this subject has been receiving increasing research attention in recent years. TRT may be a promising treatment option for hypogonadal men, older men, and patients at risk of viral infection or wasting diseases. Some contraindications and side effects may limit the application of TRT in clinical settings, including increased of hair loss (Dallob et al. 1994), infertility (Crosnoe et al. 2013), as well as some conflicting evidence on the development of CVD (Corona et al. 2011). These risks must be evaluated on an individual basis and weighed against the potential benefits to the quality of life and longevity of the patient.

Additionally, the role of androgens is complex due to crosstalk with multiple organ systems, cellular processes, and metabolism into various bioactive compounds. One of the more promising aspects of androgen action is interaction with the body’s inflammatory system. Testosterone and its metabolites may modulate various aspects of the inflammatory cascade, apoptosis, and cellular aging. Current literature provides evidence that androgens may play a significant role in decreasing multiple pro-inflammatory markers while also increasing several anti-inflammatory markers. These effects may explain why healthy androgen levels seem to attenuate disease of many organ systems and correlate with lower all-cause mortality. The impacts of aging must also be considered in this context. The process of aging has been shown to correlate with higher levels of inflammation, ROS, harmful microbiota, and senescent cells. Androgens have been shown to improve these markers, which may explain why eugonadal older men have better health outcomes and quality of life. These findings may also support the use of androgen replacement in hypogonadal older men.

Two critical features of age-related wasting are sarcopenia and osteopenia. Both conditions result in progressive loss of strength, movement ability, and quality of life. In a clinical context, these conditions lead to increased risk of immobility, dependence, and injury from daily activity. Older men with healthy androgen levels are significantly less likely to exhibit symptoms of muscular or skeletal wasting, which further supports the conclusion that TRT may be beneficial to treat and prevent cachexia in older men. Inflammation has also been shown to be a key contributor to the development of sarcopenia and osteopenia. The findings that T and DHT lead to a downstream anti-inflammatory response provide further evidence that androgen replacement could be a valuable tool to combat age-related wasting for older hypogonadal men.

The observed decline in androgen levels with age may be due to several contributing factors, including increased SHBG levels, dysfunction of the hypothalamus and anterior pituitary glands, and loss of testicular secretion. As a result, the decline in androgen levels in the aging male requires further examination and research due to its importance in a variety of diseases, as well as quality of life. Future studies should focus on clarifying the causes and effects of male hypogonadism and its impact on health. The current androgen replacement therapy protocol also requires further investigation into benefits, contraindications, and long-term safety profiles.

## Abbreviations

T: Testosterone
HPG: Hypothalamic-pituitary-gonadal
GnRH: Gonadotrophin releasing hormone
LH: Luteinizing hormone
FSH: Follicle stimulating hormone
SHBG: Sex hormone binding globulin
HSA: Human serum albumin
CBG: Corticosteroid-binding globulin
AR: Androgen receptor
DHT: 5α-Dihydrotestosterone
AREs: Androgen response elements
ERK: Extracellular signal regulated kinase
MAPK: Mitogen activated protein kinase
BMD: Bone mineral density
MPS: Muscle protein synthesis
IGF1: Insulin like growth factor 1
FA: Fatty acid
LPL: Lipoprotein lipase
ACS: Acyl coenzyme A synthetase
CVD: Cardiovascular disease
CRP: C reactive protein
EPO: Erythropoietin
TGFβ: Transforming growth factor β
TLR: Toll like receptor
TNF-α: Tumor necrosis factor α
IL: Interleukin
DC: Dendritic cells
ROS: Reactive oxygen species
Aβ: β-Amyloid
AD: Alzheimer’s disease
EAE: Experimental autoimmune encephalomyelitis
MS: Multiple sclerosis
NPCs: Neural progenitor cells
TBI: Traumatic brain injury
TRT: Testosterone replacement therapy
HIV: Human immunodeficiency virus
AIDS: Acquired immunodeficiency syndrome
GABA: γ-Aminobutyric acid
IM: Intramuscular
RCTs: Randomized control trials
COPD: Chronic obstructive pulmonary disease
LBM: Lean body mass
AAS: Anabolic androgenic steroid
BCM: Body cell mass
DHEA: Dehydroepiandrosterone
DHEA-S: Dehydroepiandrosterone-sulfate
SARS: Severe acute respiratory syndrome
ARDS: Acute respiratory distress syndrome
IFN-γ: Interferon γ
ACE2: Angiotensin converting enzyme II
ECMO: Extracorporeal membrane oxygenation
TMPRSS2: Transmembrane serine protease II
ADT: Androgen deprivation therapy
ARKO: Androgen receptor knockout
WT: Wild type
ED: Erectile dysfunction
NO: Nitric oxide
NF-κB: Nuclear factor kappa B
VCAM-1: Vascular cell adhesion molecule 1
HDL: High density lipoprotein
hs-CRP: High sensitivity C reactive protein
T2D: Type 2 diabetes
GR: Glucocorticoid receptor
LDL: Low density lipoprotein
RMR: Resting metabolic rate
AITD: Autoimmune thyroid disease
T1D: Type 1 diabetes
JAK/STAT: Janus kinase/signal transducer
ILC: Innate lymphoid cell
AP-1: Activator protein 1
SASP: Senescence-associated secretory phenotype
BTx: Botulinum toxin
StAR: Steroidogenic acute regulatory
GH: Growth hormone
mTOR: Mammalian target of rapamycin
AMPK: AMP activated protein kinase
PI3K: Phosphatidylinositol-3 kinase
